# The Clinical Thermometer

**Published:** 1881-12

**Authors:** 


### Editorial.

### THE CLINICAL THERMOMETER.

To the skillful physician the clinical thermometer is an indispensable instrument.

Its value is attested in the greater accuracy of his diagnosis; and the impartial testimony which it affords, ofttimes constitutes an important element in his prognosis.

The practice of determining the temperature of a patient by the sensation produced from contact with the surface of his body is obviously so liable to error as to be utterly untrustworthy and entirely useless except in the coarsest and most general way.

The surface-heat and the blood-heat represent distinct, and often widely different conditions, and, while the one may be an index of the other, it is frequently the case that the utmost divergence exists, and thus the physician, in seeking to ascertain the temperature of the body, is exposed to the risk of serious deception if he depends upon his own perceptions, excited through the sense of touch.

The importance, then, of the thermometer—the heat measurer—in a clinical point of view, depends upon the fact that it is a means of accuracy—that it gives to the opinions and prognostications of the physician a degree of reliability and certainty to which they could not attain without the evidence which it alone can furnish. Hence the correctness of the instrument is a question of great moment, for, unless it is absolutely true, its testimony is not only valueless, but, if accepted, disappointment and disaster are likely to ensue. It then fails in its mission, and its indications are useless—yea, worse than useless; it is a false light; a treacherous guide; a broken reed.

In view, therefore, of these facts, the establishment of a Thermometric Bureau by the Winchester Observatory of Yale college, for the purpose of testing and verifying thermometers by comparison with standard instruments, possesses special interest for medical practitioners; and the object of this article is to emphasize the importance of clinical thermometry—of reliable clinical thermometry—and to point out the precise means by which it can be secured.

Through the courtesy of Mr. Leonard Waldo, the astronomer in charge of the observatory, we are enabled to give the following difinite information.

Thermometers will be received by the observatory for comparison with the observatory standard, and certificates of comparison will be issued setting forth the necesary corrections to be made in order to compensate the deviation from the standard.

All thermometers sent for verification must have a name and number engraved upon them, and the accompanying letter of advice should contain the maker's name, the number on the instrument, the full address of the sender, with specific directions for reshipment, and fifty cents, which covers the total cost for the rectification of each clinical thermometer.

Thermometers which are not graduated on the glass stem must be of sufficiently good workmanship to insure permanence in the relative position of the index and the tube.

To make sure the safe transmission of thermometers by express they should be placed in two boxes—one within the other—and the space between the two filled with cotton. Single clinical thermometers may be carefully packed in a wooden box and sent by mail.

A thermometer sent in the manner and with the precautions above indicated, will be returned within three days of the time of its reception, with the certificate of the observer, showing the plus or minus corrections that should be applied at each one-fifth of a degree of the scale.

The express address is "Winchester Observatory of Yale College, New Haven, Connecticut." The post-office address is "Box No. 853.

It is important to remember that the inaccuracy of clinical thermometers is not always due to careless or defective manufacture, but sometimes to subsequent changes in the tube.

Mercurial thermometers increase their readings with age, and during the first year after their manufacture this increase amounts to one degree or more.

The requisite age of the tube to insure thorough seasoning, and to prevent further appreciable variation is about two years; so that an old thermometer is better than a new one.

The observatory is now verifying an average of three hundred thermometers a month, and the official report shows that the errors in their readings range from one-half to two degrees!

This bare statement, we doubt not, will suffice to arouse our readers to a recognition of the necessity for prompt attention to this subject, and cause them to take advantage of the opportunity which the Winchester Observatory offers.

